# The Use of Olfactory and Visual Cues in Host Choice by the Capsid Bugs *Lygus rugulipennis* Poppius and *Liocoris tripustulatus* Fabricius

**DOI:** 10.1371/journal.pone.0046448

**Published:** 2012-12-03

**Authors:** Fiona J. H. Wynde, Gordon R. Port

**Affiliations:** School of Biology, Ridley Building, University of Newcastle upon Tyne, Newcastle upon Tyne, United Kingdom; Max Planck Institute for Chemical Ecology, Germany

## Abstract

*Lygus rugulipennis* Poppius and *Liocoris tripustulatus* Fabricius (Heteroptera: Miridae) are pests of glasshouse cucumber and sweet pepper crops respectively. *L. rugulipennis* has a wide range of foodplants, but *L. tripustulatus* is specialised with very few food plants. We report behavioural assessments to investigate whether either species exhibits a preference for salad over wild hosts, and whether the role of olfaction and vision in response to cues from host plants can be distinguished. Olfactory responses to leaves were tested in choice chambers. *L. rugulipennis* was presented nettle (wild host) and a salad leaf of cucumber or sweet pepper, where the salad leaves had higher nitrogen content. *L. tripustulatus* was tested with nettle and sweet pepper of two different nitrogen contents. Female *L. rugulipennis* spent more time on the cucumber salad host, and chose it first most often, but males showed no preference. Neither sex discriminated between sweet pepper or nettle leaves, but males made more first contacts with sweet pepper. Neither sex of *L. tripustulatus* discriminated between sweet pepper and nettle leaves when the sweet pepper had higher nitrogen. When the plant species contained equivalent nitrogen both sexes spent more time on nettle. There was no difference in first choice made by either sex. When visual stimuli were available, and leaves had equivalent nitrogen, *L. rugulipennis* showed no preference and *L. tripustulatus* preferred nettle leaves. We conclude that the generalist *L. rugulipennis* has the ability to use remote olfactory cues for host choice whereas the specialist *L. tripustulatus* relies mainly on contact chemosensory and gustatory cues.

## Introduction

Capsid bugs (Heteroptera: Miridae) are a sporadic pest of horticultural crops including certain protected salad crops [1]–[3]. They are rarely abundant pests, but feeding and damage from oviposition can cause important economic losses. Demand for blemish-free produce means that scarred, holed and misshapen fruit are unacceptable. Feeding damage to the growing tips and leaves of glasshouse crops is also a problem as plant growth is affected.

The tarnished plant bug, *Lygus rugulipennis* Poppius is a pest of protected cucumber (*Cucumis sativa* L., Cucurbitaceae) crops. The common nettle capsid, *Liocoris tripustulatus* Fabricius is a pest of protected sweet pepper (*Capsicum annuum* L., Solanaceae) crops [2]. Both species have caused problems in glasshouses in the UK. Before the introduction of integrated pest management (IPM), capsids were probably controlled by chemical pesticides used against the other major glasshouse pests. Reduced chemical use associated with IPM has allowed capsids to survive and breed in glasshouse crops [2].

*L. rugulipennis* is also an important field crop pest in mainland Europe. Previous studies have focused on their pest status on crops of lucerne [4], wheat [5], [6], sugar beet [7], cabbage [8], maize [9], peaches [10] and hops [11]. Damage to protected crops, i.e. those grown under glass or plastic, has not been extensively reported and, in Europe, reports have been limited to cucumbers in Britain [2] and Finland [1]. In British Columbia, Canada, *Lygus* spp. are a sporadic pest of both cucumber and sweet pepper glasshouse crops [3].

There have been few reports in the literature of pest problems caused by *L. tripustulatus*. Damage to protected crops has occurred in sweet peppers in the UK [2], and in sweet pepper [12], and paprika [13], [14] in mainland Europe. Šedivý & Fric [11] found *L. tripustulatus* in hop yards in the Czech Republic, however the relative economic importance of this capsid species compared with others found on the crop was not investigated. *L. rugulipennis* and *Lygocoris lucorum* (Meyer-Dür) were the dominant species in the crop which may indicate limited damage by *L. tripustulatus*.

Although both *L. rugulipennis* and *L. tripustulatus* show similar traits in glasshouse crops, they exhibit different life histories and host choice in the wild. *L. rugulipennis* is polyphagous, with over 400 species [sic] from 57 families recorded as host plants in Europe [15]. Of these, there are 51 British records from 18 families [15]. *L. tripustulatus* has been recorded almost exclusively from nettle (*Urtica dioica* L, Urticaceae) [16], although there are records from the Labiate family: white dead nettle (*Lamium album* L.) (feeding observed, pers. obs.) and mint (*Mentha* sp.) [17]. These life history traits, may reflect the use of different cues for locating host plants by a generalist and a specialist species of phytophagous insect.

Successful control of capsids within existing, and possible future, IPM programmes requires detailed knowledge of the pests' biology and ecology. Capsid bugs may overwinter in the glasshouses, but lack of pest damage on early crops suggests that this is unlikely. Alternatively they may be attracted into the glasshouse crops by plant volatiles from the salad crops. This may occur at a time when their wild hosts are nutritionally poorer than the salad hosts (e.g. [18]). Optimal foraging theory predicts that a greater time should be spent on more rewarding patches of food [19]. The study presented here uses behavioural assessments to investigate whether either species exhibits a preference for salad over wild hosts, and whether the role of olfaction and vision in response to cues from host plants can be explained. The experiments demonstrated differences exhibited by each species.

## Materials and Methods

### Ethics statement

No specific permits were required for the described field studies, with collections being made from University-owned land which is not designated (protected). The collections did not involve endangered or protected species.

### Insects

#### *Lygus rugulipennis*

Cultures of *L. rugulipennis* were maintained in the laboratory at 21°C (±2°C), and L16∶D8. Adults and nymphs were reared on sprouting potatoes with *ad lib* additions of green or runner beans. During the field season (July to November) cultures were regularly supplemented with wild caught individuals. Before each trial, 5^th^ instar nymphs were isolated from cultures, kept individually in Petri dishes, and maintained at 21°C (±2°C) until adult emergence. Individuals were provided with moist cotton wool, a piece of tissue paper, and a fresh green bean each day. Rearing pots were checked twice daily for the presence of emerged adults and the food withdrawn. Adults were starved for 24–48 h before choice tests. All adults were used in tests within 60 hours of emergence. Sex of individuals was determined by examination of the abdomen [20].

#### *Liocoris tripustulatus*

Second generation adult *L. tripustulatus* were collected during July and August 2000 from Close House Field Station (University of Newcastle (Grid Reference NZ 1265). Individuals were kept separately in Petri dishes with a piece of tissue paper and moist cotton wool. They were starved immediately upon collection from the field for –at least 24 hours before the experiment. All adults were used in experiments within 60 hours of collection. Sex was determined as for *L. rugulipennis*.

### Plants

Sweet pepper (*C. annuum* var. Worldbeater), cucumber (*C. sativa* var. *sativa* Improved Telegraph) and nettle (*Urtica dioica* L., Urticaceae) plants were grown in pots (15–20 cm diameter) in a glasshouse. All plants were given 20∶10∶10 NPK (granules: 20% N, of which 9% was nitrate nitrogen and 11% ammonical nitrogen (Kelmire Ince Ltd., Chester)) fertiliser treatments (applied at a rate of 21 g/m2) to prevent yellowing of leaves in early development. Peppers used in one of the treatments (treatment 2) were also given an application of nitrogen (granules 21 g/m^2^: 33.5% N ammonium nitrate) four days before the experiment. This application of nitrogen granules resulted in a lower total nitrogen content in the leaves of these plants compared with the plants given only the 20∶10∶10 fertiliser treatment, therefore the two treatments are referred to as ‘high nitrogen’ (only 20∶10∶10) and ‘low nitrogen’ (20∶10∶10 plus 33.5% ammonium nitrate) throughout. Plants grown in excess nitrogen usually exhibit increased growth and higher nitrogen content, however, it is likely that the ratio of essential elements was disturbed with the addition of ammonium nitrate, leading to lower total % nitrogen levels [21].

### Determination of nitrogen levels in experimental plants

Leaves were taken for trials from a similar position on each of the experimental plants, freeze-dried (for 144 h) and ground to a powder. Nitrogen content was determined using automatic nitrogen carbon analysis (Roboprep CN Biological Sample Converter (Europa Scientific)). This technique measures the total nitrogen content of the sample material as a percentage of the total leaf mass.

### Olfactory experiments

In order to assess the role of olfactory stimuli in host choice experiments were done in choice chambers placed within an incubator (Sanyo MIR-253), from which light was excluded. In preliminary trials with a linear track olfactometer and a Pettersson four-way olfactometer, when air was drawn through the apparatus *L. rugulipennis* and *L. tripustulatus* did not exhibit normal behaviour consistent with that observed in cultures and in the field. Therefore a modified choice chamber, with no air flow, was designed where the more usually observed movement and antennation behaviours were present. The trials were carried out under constant temperature (21°C±2°C). Individual adults were introduced to a rectangular choice chamber (225 mm×120 mm×85 mm), lined with paper towel (replaced after each trial). Single, sized-matched (by eye), leaves of the test species were placed at either end of the chamber and adults were introduced to a central dish (40 mm diameter, 50 mm height). The species of leaf placed at each end of the chamber was alternated in consecutive trials for each sex. Activity was recorded over a period of one hour using a video camera (Baxall CD9242/IR), infra-red illumination, and a Panasonic AG-6040 time-lapse video recorder placed directly above the choice chamber. The majority of insects are not thought to be able to detect wavelengths above around 650 nm; red in the visible spectrum [22], and the insects used in this study showed no obvious response to the presence of the infra-red illumination.

The trials were carried out between the following dates in 2000:

*Lygus rugulipennis* cucumber versus nettle - 8^th^ March to 16^th^ July

*Lygus rugulipennis* pepper versus nettle - 9^th^ May to 10^th^ November

*Liocoris tripustulatus* ‘high nitrogen’ pepper versus nettle - 1^st^ August to 25^th^ August

*Liocoris tripustulatus* ‘low nitrogen’ pepper versus nettle – 24^th^ August to 8^th^ September

Between 24^th^ February and 5^th^ April 2001 *L. rugulipennis* trials with cucumber and nettle were repeated (10 males and 10 females). There was no statistical difference between these data and the corresponding trials carried out in 2000. Therefore, data from both years were pooled for analysis.

Results of the *Lygus rugulipennis* pepper versus nettle trial were analysed using a General Linear Model to test effects of date, time of day and position of leaf in the choice chamber. This was the only data set where it was possible to transform data to a normal distribution. Otherwise, a non-parametric statistical test was used.

### Behavioural assessments

#### Host encounter behavior

A contact was deemed to have commenced when the capsid touched a leaf with its antennae or proboscis, and ended when it moved away from the leaf.

#### First contact

If the insects are making a remote host choice based on olfaction alone the preferred species would be expected to be the first choice made in a trial. The first contact made in each trial was noted.

#### Time spent on each leaf

The proportion of total contact time with each leaf during a trial was noted.

#### Combined stimuli (olfaction and vision) experiments

In order to assess the role of olfactory and visual stimuli in host choice, these experiments were done in choice chambers where the insects had both available stimuli. All trials were carried out in a Sanyo MIR-253 incubator (Sanyo Electric Co., Ltd., Japan) at 21±2°C between 0800 and 1900. Natural light was allowed into the incubator through a clear Perspex panel. A choice chamber of clear perspex (350 mm×220 mm×440 mm) was used for the experiments. Cuttings were taken from experimental plants so that there was an approximately equal leaf area, with at least three terminal leaves on offer to the capsids. Capsids are usually found feeding on the terminal parts of their wild plant hosts [1]. Cuttings were pushed through a layer of horticultural fleece into a water supply. The fleece formed a floor on which the capsids were placed. Individuals were introduced into the choice chamber and left undisturbed for one hour, after which, their position (on an experimental plant, or having not made a choice) was noted. The sensitivity of the capsids to any movements external to the choice chamber prevented continual monitoring. However, preliminary observations showed that they were likely to remain on a chosen host for the duration of the experiment. *Lygus rugulipennis* was given a choice between cucumber and nettle (n = 20 males, 20 females) and *Liocoris tripustulatus* a choice between pepper and nettle (n = 20 males, 20 females).

## Results

### Determination of nitrogen levels in experimental plants

**Figure 1:**
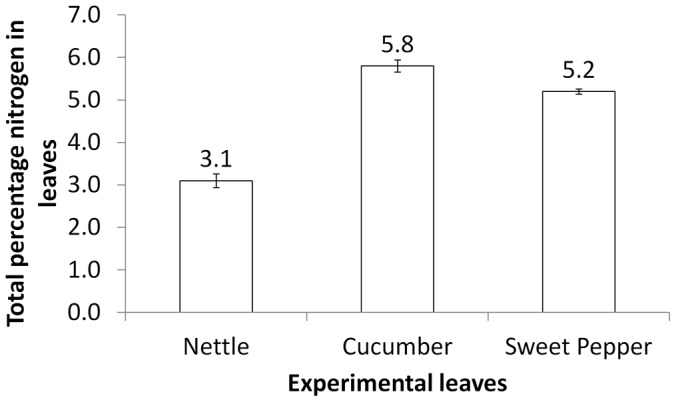
Total percentage nitrogen in experimental leaves offered to *Lygus rugulipennis.* *Post hoc* comparisons (Fisher's pairwise comparisons) showed that the level of nitrogen in the salad leaves differed significantly to the nettle leaves but not to each other.

**Figure 2:**
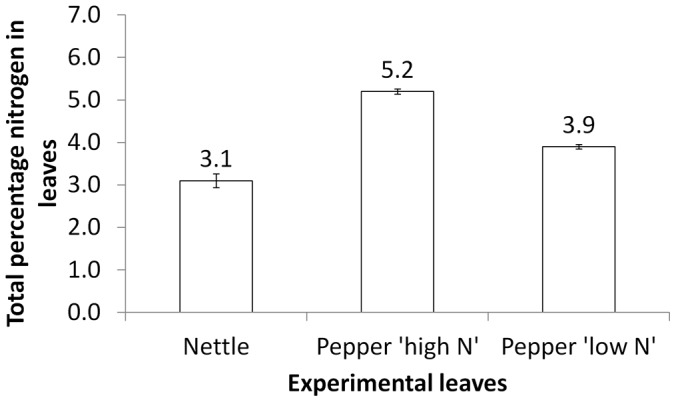
Total percentage nitrogen in experimental leaves offered to *Liocoris tripustulatus.* *Post hoc* comparisons (Fisher's pairwise comparisons) showed that the nettle leaves had significantly lower total percentage nitrogen than ‘high nitrogen’ pepper. There was no significant difference in total percentage nitrogen between nettle and ‘low nitrogen’ pepper leaves.

Nitrogen data were arcsine transformed before analysis and compared using a one-way analysis of variance. In tests with *L. rugulipennis* the median (n = 4) percentages of total nitrogen in leaves were: nettle = 3.1, cucumber = 5.8 and pepper = 5.2 (F = 104.18, d.f. = 2, P<0.001). *Post hoc* comparisons (Fisher's pairwise comparisons) showed that the level of nitrogen in the salad leaves differed significantly to the nettle leaves but not to each other (Figure 1). Leaves used in the *L. tripustulatus* trials had median (n = 4) percentages of total nitrogen of: nettle = 4.2, pepper = 5.2 (subsequently referred to as ‘high nitrogen’) and pepper = 3.9 (subsequently referred to as ‘low nitrogen’) (F = 73.56, d.f. = 2, P<0.001). *Post hoc* comparisons showed that the nettle leaves had significantly lower total percentage nitrogen than ‘high nitrogen’ pepper. There was no significant difference in total percentage nitrogen between nettle and ‘low nitrogen’ pepper leaves (Figure 2).

Plants used in the olfaction+vision experiments did not show any significant differences in total nitrogen content (median (n = 4): cucumber = 4.02%, nettle = 4.72%, sweet pepper = 4.47%).

### Olfactory Experiments

#### *Lygus rugulipennis*

Females showed a significant preference for cucumber (salad host) over nettle (wild host) (Table 1). They chose cucumber leaves first in significantly more trials than nettle leaves and spent longer in contact with cucumber leaves Males did not exhibit any preference between cucumber and nettle leaves (Table 1). Mean time to first contact with leaves was 1118 s±117 s (females) and 1216 s±173 s (males). The cucumber leaves had higher nitrogen content relative to the nettle leaves.

**Table 1:** *Lygus rugulipennis* offered a choice of cucumber (salad host) and nettle (wild host) leaves in a darkened choice chamber – summary of results.

Behaviour	Sex	Cucumber	Nettle	Result^1^	Conclusion
Median total time spent on each leaf as proportion of total contact time (sec)	F	1.0	0.0	W = 386.0, P<0.01	Females discriminate
	M	0.77	0.23	W = 263.0, ns	Males do not discriminate
Number of first contacts	F	23	7	?^2^ = 7.5, P<0.01	Females discriminate
	M	17	13	?^2^ = 0.3, ns	Males do not discriminate

1 n = 30 for each sex. Mann-Whitney test used to compare median time on leaf; Chi square test used to compare number of first contacts; ns = not significant.

Females showed a significant preference for cucumber (salad host) over nettle (wild host). They chose cucumber leaves first in significantly more trials than nettle leaves. Males did not exhibit any preference between cucumber and nettle leaves.

In the choice tests with nettle and sweet pepper males of *L. rugulipennis* made first contact with sweet pepper on significantly more occasions than nettle (Table 2). This was the only preference exhibited by male or female *L. rugulipennis* in this set of experiments. The sweet pepper leaves had higher nitrogen content relative to the nettle leaves. Mean time to first contact with leaves was 850 s±181 s (females) and 1061 s±199 s (males). Application of a general linear model (GLM) (difference in time spent on each leaf (minutes) versus sex) confirmed no significant difference in contact time between sexes (F = 0.38; P = 0.54) and also showed that there were no significant effects of date of experiment (F = 2.24; P = 0.144), time of day (F = 0.00; P = 0.975) or position of leaf in the choice chamber (F = 0.48; P = 0.495) on the results. This was the only set of olfactory choice data for which a GLM was possible.

**Table 2:** *Lygus rugulipennis* offered a choice of sweet pepper (salad host) and nettle (wild host) leaves in a darkened choice chamber – summary of results.

Behaviour	Sex	Sweet pepper (‘low’ nitrogen)	Nettle	Result^1^	Conclusion
Median total time spent on each leaf as proportion of total contact time (sec)	F	0.33	0.67	W = 64.0, ns	Females do not discriminate
	M	0.59	0.41	W = 126.5, ns	Males do not discriminate
Number of first contacts	F	6	14	?^2^ = 2.45, ns	Females do not discriminate
	M	16	3	?^2^ = 7.58, P<0.01	Males discriminate

1 n = 30 for each sex. Mann-Whitney test used to compare median time on leaf; Chi square test used to compare number of first contacts; ns = not significant.

In the choice tests with nettle and sweet pepper males of *L. rugulipennis* made first contact with sweet pepper on significantly more occasions than nettle This was the only preference exhibited by male or female *L. rugulipennis* in this set of experiments.

#### *Liocoris tripustulatus*

When offered a choice of nettle and sweet pepper (‘high nitrogen’), ie when the pepper had significantly higher nitrogen content than nettle, there were no preferences in total contact time or species of first choice exhibited by either sex (Table 3). Time to make first choice differed significantly with females taking longer: mean 1605 s±134 s (females) and 1143 s±141 s (males) (ANOVA: F = 4.0, P = 0.02).

**Table 3:** *Liocoris tripustulatus* offered a choice of sweet pepper (salad host) and nettle (wild host) leaves when the salad host had higher % nitrogen content in a darkened choice chamber – summary of results.

Behaviour	Sex	Sweet pepper (‘high’ nitrogen)	Nettle	Result^1^	Conclusion
Median total time spent on each leaf as proportion of total contact time (sec)	F	0.05	0.95	W = 146.5, ns	Females do not discriminate
	M	0.19	0.81	W = 191.5, ns	Males do not discriminate
Number of first contacts	F	10	20	?^2^ = 2.7, ns	Females do not discriminate
	M	13	7	?^2^ = 0.8, ns	Males do not discriminate

1 n = 30 for each sex. Mann-Whitney test used to compare median time on leaf; Chi square test used to compare number of first contacts; ns = not significant.

When offered a choice of nettle and sweet pepper when the pepper leaves had higher nitrogen content than nettle leaves, there were no preferences exhibited (Table 3).

When there was no difference in nitrogen content of the nettle and sweet pepper (‘low nitrogen’) leaves both sexes spent significantly more time in contact with nettle leaves (Table 4). There was no difference in the first leaf chosen in any of the trials with *L. tripustulatus* and no difference in time to make first choice: mean 1457 s±132 s (female) and 1601 s±185 s (males).

**Table 4:** *Liocoris tripustulatus* offered a choice of sweet pepper (salad host) and nettle (wild host) leaves with the same % nitrogen content in a darkened choice chamber – summary of results.

Behaviour	Sex	Sweet pepper (‘low nitrogen’)	Nettle	Result^1^	Conclusion
Median total time spent on each leaf as proportion of total contact time (sec)	F	0.2	0.98	W = 103.0, P<0.01	Females discriminate
	M	0.01	0.99	W = 133.0, P<0.05	Males discriminate
Number of first contacts	F	10	20	?^2^ = 2.7, ns	Females do not discriminate
	M	11	19	?^2^ = 1.63, ns	Males do not discriminate

1 n = 30 for each sex. Mann-Whitney test used to compare median time on leaf; Chi square test used to compare number of first contacts; ns = not significant.

When there was no difference in nitrogen content of the nettle and sweet pepper leaves both sexes spent significantly more time in contact with nettle leaves. There was no difference in the first leaf chosen in any of the trials with *L. tripustulatus*.

### Olfactory and visual stimuli experiments

#### *Lygus rugulipennis*

Male and female *L. rugulipennis* did not exhibit any preference when offered cucumber and nettle cuttings (leaves with no significant differences in total percentage nitrogen content). Results for males and females were identical (number of individuals on cucumber after one hour = 11, number on nettle = 9, χ^2^ = 0.05, ns).

#### *Liocoris tripustulatus*

Both male and female *L. tripustulatus* preferred cuttings of their wild host, nettle, to sweet pepper (leaves with no significant differences in total percentage nitrogen content). Results for males and females were identical (number of individuals on sweet pepper after one hour = 5, number on nettle = 15, χ^2^ = 4.05, P<0.05).

## Discussion

The two capsid species used in this study have different life history traits – *L. rugulipennis* is polyphagous, *L. tripustulatus* is oligophagous. The species exhibited different behaviours when presented with a choice of host leaves which differed in their relative nitrogen contents.

### *Lygus rugulipennis* – the generalist

When presented with plant cuttings of species that are recorded as salad (cucumber) and wild (nettle) hosts, with equivalent nitrogen content there was no preference exhibited by either sex. In contrast, when cucumber leaves of relatively higher nitrogen content were offered in a test against nettle leaves, female *L. rugulipennis* exhibited a preference for cucumber leaves (more first contacts and greater proportion of time). Males did not show any significant preference in first contact or in total amount of time in contact with each plant species. These trials were carried out in absence of visual cues, meaning the insects were reliant on olfaction, or contact cues following random trials. Although the volatiles will have mixed over the period of the experiment in the open chamber, the significant difference in first contact made indicates that this choice could have been made using olfactory cues.

When offered an alternative salad host (sweet pepper) which also had higher relative nitrogen than the nettle leaves, but which has not been recorded as a host plant for this species in the UK, different choices were exhibited. Females showed no preference between the leaves, making equal numbers of first contacts and spending similar amounts of time in contact with each. Males showed no difference in total contact time, but did make significantly more first contacts with sweet pepper.

### *Liocoris tripustulatus* – the specialist

When *L. tripustulatus* adults were given a choice between cuttings of a known wild (nettle) and salad (sweet pepper) host which had equivalent levels of relative nitrogen, both sexes preferred the wild host, nettle. Individuals used in this trial were collected from the wild and will therefore have experienced nettle prior to the trials. This reflects the commercial situation where the adults infesting the glasshouses are second generation and will therefore have experienced nettle as a wild host.

In tests carried out in a darkened choice chamber, both sexes of *L. tripustulatus* again demonstrated a preference for their wild host (nettle) when the salad host offered contained equal levels of total nitrogen. Both sexes spent more time in contact with the nettle leaves, but did not exhibit any significant difference in the host that first contact was made with. However, when the sweet pepper host contained higher levels of nitrogen relative to the nettle leaves there was no difference in total contact time for either sex.

One disadvantage of the choice chamber used was the lack of air flow, a constraint imposed by the unusual behaviour of the insects when confined in chambers with an air flow. With the closed chamber there is a risk that plant volatiles will saturate the air-space. However, two of the trials showed non-random first choice suggesting that saturation of the air-space was not a significant issue. The experimental set up did not allow direct testing of repulsion versus attraction to volatiles.

### Does total level of nitrogen in host leaves influence choice?

Male and female *L. rugulipennis* demonstrated differences in response when given a choice of cucumber leaves (higher nitrogen content) and nettle leaves, with females exhibiting more initial contacts with cucumber leaves and spending more time on them. Other work indicates that nitrogen availability is a limiting factor in insect growth [23], and Holopainen &Varis [15] suggest that the nitrogen content of the host plant is the *key factor* in determining the host plant selection of *L. rugulipennis*. Indeed, oviposition rate and subsequent growth rate of nymphs on pine seedlings was positively related to increased nitrogen content [24]. Feeding on the most nitrogen rich source of host plant in order to prepare for reproduction was shown to be the case in another capsid species, *Leptopterna dolabrata* (L.) by McNeill [18]. Carnivory and cannibalism have been observed for both *L. rugulipennis* and *L. tripustulatus* in the present study, traits that often are associated with nitrogen being an important factor in their diet [25]. However, when offered a choice of nettle leaves and an alternative nitrogen rich salad host (sweet pepper), this preference by females was not repeated, and it was the males that exhibited the only preference in these trials, of first initial contact with sweet pepper leaves, having made a remote preference based on plant volatiles. There was no evidence of increased residence time on either leaf species despite the higher nitrogen content of sweet pepper. *L. rugulipennis* have not reported as a pest of sweet peppers, although they will feed on this species in the laboratory (*pers. obs.*).

When *L. tripustulatus* are offered a salad leaf with relatively higher nitrogen than their wild host they exhibited an equal preference between wild and salad hosts, in contrast to results when nitrogen content of both hosts was equal. It is possible that higher level of nitrogen available in the salad crop meant that these leaves were worth sampling for longer, overcoming a strong preference for the usual wild host, but not becoming the preferred choice. ‘Induction of preference’, where individuals increase their fidelity to a plant that has already been experienced and prefer this over a new host has been most often demonstrated in Lepidoptera, but also in Heteroptera [26]. Individuals used were taken from the field and therefore had experience of nettles. As the incidents of pest attack on glasshouse peppers have occurred mainly in August and involve the year's new generation of adults, the experiments in this study reflected this aspect of the field situation. The choice tests evaluated whether pepper offered a greater enticement to choose these leaves over the familiar nettles leaves. There was some evidence that when sweet pepper had higher levels of nitrogen the significant preference exhibited for the wild host, when relative nitrogen content was equal, was mediated. In the field this could result in *L. tripustulatus* abandoning their wild hosts as they decline in quality later in the season.

### Mechanisms of host choice

Results from trials with *L. tripustulatus* did not demonstrate any difference in the first choice made within a darkened choice chamber, although when nitrogen content of the host leaves were equal they displayed a preference for nettle. This suggests that preference was based on cues gained by contact chemoreception following random movement, rather than from remote olfactory cues. In comparison, both sexes of *L. rugulipennis* in this trial exhibited a non-random 1^st^ choice, indicating that an initial decision in these trials could be based on remote cues.

However, if plant quality, based on nitrogen levels, is being detected remotely this must be through an alteration of plant volatiles. Bruce *et al*. [27] propose that the ratio of volatiles emitted from plant leaves is a vital component of any olfactory signal, rather than taxonomically specific compounds. They present supporting evidence from Visser & Ave [28] who showed that subtly altered ratios of green leaf volatiles switched off the attractiveness of the host plant of the Colorado potato beetle. It may be that total nitrogen content of the leaves in the present study is influencing, and therefore being detected, via changes in ratios of green leaf volatiles, but the mechanism by which this could happen is unknown. It is known that volatiles emitted by some plant species change in blend proportions over time and these often indicate a physiological change in the host plant, reflecting a difference in host suitability or growth stage (eg [29]).

Chinta *et al.* [30] demonstrated that *Lygus* bugs will respond to plant volatiles in electroantennogram (EAG) studies. They found that *L. lineolaris* responded differently to some of the individual chemicals from their host plants detected by the olfactory receptors on their antennae. Electroantennogram responses (magnitude of depolarisation) to the green leaf volatiles (E)-2-hexenal and (E)-2-hexenol, and for geraniol, were significantly greater for females than for males. However, this significant difference in response by males and females was not typical for other chemicals, with the majority of responses showing no difference between males and females. A possible role of these chemicals in host orientation is discussed [30]. Again, in laboratory experiments, Williams *et al*. [31] found that female *L. hersperus* were attracted towards several plant volatiles in a y-tube olfactometer, however it was the males that exhibited greater antennal responses in the EAG trials associated with the study. Frati *et al*. [32] also showed EAG responses to plant volatiles in *L.* rugulipennis. Both sexes gave electroantennogram responses to green leaf volatiles from undamaged plants and to methyl salicylate and (E)-β-caryophyllene emitted by *Lygus*-damaged plants, suggesting that these compounds may be involved in colonization of host plants. Chen *et al*. [33] showed that female *L. lucorum* were more sensitive to plant volatiles and males were more sensitive to analogues of sex pheromones, using EAG. EAG studies of the responses of *L. rugulipennis* to individual volatiles from cucumber (and pepper) leaves may reveal differences between male and female responses.

Previous studies have identified individual volatiles from the species used in this trial which may contribute to attraction. Anderson and Metcalf [34] identified indole in the flowers of *Cucurbita maxima* (a squash) as the single component which was highly active in EAG bioassays of the cucumber leaf beetle (Chrysomelidae: *Diabrotica undecimpunctata howardi*). Field trials confirmed the potential attraction of this compound to two further species of Chrysomelid beetles (*D. virgifera virgifera* and *Acalymma vittatum*) although not to *D. u. howardi*, despite the strong EAG response. Baited traps revealed seasonal variation in sex ratio and abundance of *D. v. virgifera* in the traps.

Beyond this evidence of antennal response to plant volatiles, Frati *et* al [35] showed a behavioural response to damaged plants. They found that in an olfactometer, females were attracted to healthy plants, but didn't react to plants damaged by oviposition or by feeding, if there were no other conspecifics present. In a wind tunnel their results showed that both males and females would move towards damaged plants, although the presence of conspecifics only enhanced the females' response. Presence of eggs on the plant reduced the response. They propose that conspecifics on damaged plants are acting as pioneers and indicating a suitable new food source, whereas eggs indicate potential competition as the host has already been exploited. The evidence from the current study suggests that olfaction can play a part in the detection of host plant leaves by females in a laboratory situation, alongside contact chemoreception, however we do not know how presentation of mechanically wounded plant material, as opposed to wounding by conspecifics) affected the behaviour of the insects.

The combination of visual and olfactory responses of the North American tarnished plant bug *Lygus lineolaris* (Palisot de Beauvois) to its host plants have been investigated in a number of studies (e.g. [30], [36], [37]). It is thought that *L. lineolaris* uses a combination of visual and olfactory cues to locate its host plants. It is likely that the Palearctic species, *L. rugulipennis*, uses similar cues for host plant selection. The specific role of vision in the host choice of *Lygus* bugs remains undetermined [37].

Both sexes of *Liocoris* spent more time in contact with the nettle leaves, but did not exhibit any significant difference in the host that first contact was made with. This suggests that the insects were not making their initial choices based on remote olfactory cues, rather that contact chemosensory cues following random movement determined the outcome. There is no evidence in the literature on the mechanism of host choice in this species.

### Implications for commercial glasshouse salad crops

Introduction of new potential host plants to an area, such as those grown in agricultural or horticultural situations have frequently resulted in new insect-plant relationships [38]. Polyphagous species such as *L. rugulipennis* will often readily colonise new introduced species [38].

Sweet pepper has not suffered attack by *L. rugulipennis* despite being grown in glasshouses adjacent to cucumber crops at some sites. In the choice tests males made more first contacts with sweet pepper than with nettle. The reason for this result is not clear, and no other indications of preference were exhibited for either species by either males or females. Nor did sweet pepper volatiles repel the insects as might have been expected from the lack of pest damage by *L. rugulipennis* in the sweet pepper glasshouse crops. However, it is possible that the amount of chemical cue available, as determined by the weight of the sample, may have had an effect and that, at higher levels of volatile, the capsids may be repelled. This study provides previously unreported evidence that *L. rugulipennis* will feed on sweet pepper.

The reasons for the specialist capsid, *L. tripustulatus*, forming a new host association is less clear. If greater nitrogen levels in salad crops are sufficient to overcome induction of preference in *L. tripustulatus* this may have greater impact at times of year when the wild nettles are getting older and have less new growth. A similar situation is described by Beerwinkle and Marshall [39] who found that cotton fleahoppers (Miridae) exhibited a preference for odours from their wild hosts over cotton, on which they are a commercial pest. Their wild hosts include a member of same plant family as nettles (*Monarda punctata* Lamiaceae). Actual volatile chemicals associated with attraction were not identified in this experiment. *Mondarda* is a spring weed. Newly hatched nymphs feed and complete one or more generations on this host. As the season progresses, the wild hosts mature and become less attractive compared with cotton as it develops new flower buds. If a similar situation could be confirmed by investigation for *L. tripustulatus* it may suggest a possible management option for glasshouse owners of regular cutting of nettles in the vicinity of the glasshouses to maintain new growth. Researchers in the UK have identified the correct ratio of sex-pheromones to demonstrate attraction in male *L. rugulipennis,* although intraspecific variation in response was present. However, they were not able to demonstrate the same response for *L. tripustulatus*. Determining the host plant specific volatiles for this species in particular could be an important factor in the ongoing development of effective traps (unpublished data).

The only other feeding records of *L. tripustulatus* from wild hosts are from two species within the same plant family, Labiatae. The new salad host association is with a member of the Solanaceae. Bearing in mind this new association, it is curious that there have never been records of this capsid as a pest of tomato *Lycopersicon esculentum* Mill. (*L. lycopersicum* (L.) Karsten) (also Solanaceae) which is a frequent glasshouse crop in Humberside (occurring in glasshouses adjacent to a capsid-affected sweet pepper crop at one site).

## Conclusions

This study has shown that when *L. rugulipennis* females use olfaction to detect host plant volatiles they choose their current salad host, cucumber, in preference to their wild host in an experimental situation. The results also show that this species will feed on sweet pepper in the laboratory and so may have the potential to become a pest of sweet pepper crops in the future This species may be able to detect plant cues from a commercial crop from a distance.

*L. tripustulatus* appear to need other cues in addition to olfaction to detect their hosts. Where there was no difference in the nitrogen content of the leaves nettle was the preferred host. However, when sweet pepper leaves of greater nitrogen content than the nettle leaves were offered no preference was exhibited. This suggests that the basic preference for nettle may be mediated by relative nitrogen content. If a salad crop has higher nitrogen content this species may start to select it as a host at times when its wild host is of lower quality.
